# Oogenesis of Hematophagous Midge *Forcipomyia taiwana* (Diptera: Ceratopogonidae) and Nuage Localization of Vasa in Germline Cells

**DOI:** 10.3390/insects11020106

**Published:** 2020-02-05

**Authors:** Szu-Chieh Wang, Yung-Hao Ching, Preethi Krishnaraj, Guan-Yu Chen, Anna Shiny Radhakrishnan, Hsien-Min Lee, Wu-Chun Tu, Ming-Der Lin

**Affiliations:** 1 Department of Molecular Biology and Human Genetics, Tzu Chi University, Hualien 97004, Taiwan; paper90312@yahoo.com.tw (S.-C.W.); yching@gms.tcu.edu.tw (Y.-H.C.); preethisixnov@gmail.com (P.K.); shinejas4@gmail.com (A.S.R.); 2 Department of Medical Research, Hualien Tzu Chi Hospital, Hualien 97002, Taiwan; 3 Department of Life Science, Tzu Chi University, Hualien 97004, Taiwan; 104711131@gms.tcu.edu.tw; 4 Graduate Institute of Biotechnology, Central Taiwan University of Science and Technology, Taichung 40601, Taiwan; hmlee@ctust.edu.tw; 5 Department of Entomology, National Chung Hsing University, Taichung 40227, Taiwan; wctu@dragon.nchu.edu.tw; 6 Institute of Medical Science, Tzu Chi University, Hualien 97004, Taiwan

**Keywords:** bloodsucking midge, *Forcipomyia*, oogenesis, Vasa, nuage

## Abstract

*Forcipomyia taiwana* is an irritating hematophagous midge that preferentially attacks humans and affects leisure industries in Taiwan. Understanding the female reproductive biology of such pests would facilitate the development of pest control strategies. However, knowledge about oogenesis in the genus *Forcipomyia* is unavailable. Accordingly, we examined the ovariole structure and features of oogenesis in terms of the oocyte and the nurse cell. After being blood-fed, we observed a high degree of gonotrophic harmony—the synchronization of developing follicles. The follicle of the *F. taiwana* has only one nurse cell connected to the oocyte, which is distinct among hematophagous midges. In the nurse cell, we identified the perinuclear localization of the germline marker, Vasa. The Vasa localization is reminiscent of the nuclear envelope-associated nuage observed by electron microscopy. To determine whether *F. taiwana* Vasa (FtVasa) is an authentic nuage component, we produced transgenic flies expressing FtVasa in the female germline and proved that FtVasa was able to be localized to *Drosophila* nuage. By characterizing the oogenesis and Vasa expression in the germline cells of *F. taiwana*, this study extends knowledge about the female reproductive biology of hematophagous midges.

## 1. Introduction

*Forcipomyia taiwana*, first reported in 1913 by Shiraki [1], is a major nuisance hematophagous midge in the urban and suburban areas of Taiwan. The prevalence of this midge was reported to be seasonal from April to September, and massive swarms of such midges can be observed in regions with cultivations and moderately moist conditions [2]. During the daytime, the female midge preferentially attacks exposed human body parts to obtain blood meal for egg production [3]. The preference of *F. taiwana* for human blood was demonstrated by the enhancement of its feeding efficiency on a New Zealand rabbit after the application of human sweat on the rabbit’s skin [4]. Because *F. taiwana* bites can cause severe allergic responses in sensitive individuals [5,6], the presence of *F. taiwana* engenders unfavorable conditions for outdoor activities and affects tourism economically [2].

The prevalence of *F. taiwana* relies on its remarkable capacity to adapt to the human environment and generate a large number of offspring. Therefore, the knowledge of ovarian development and oogenesis would provide us with crucial information on how the eggs are efficiently produced after a blood meal and could be helpful for developing a strategy for population control. Although approximately 5000 species of hematophagous and nonhematophagous midges are included in the family Ceratopogonidae [7,8], knowledge about their ovarian structure and oogenesis is limited to a few reports on the genus *Culicoides* [9,10]. Specifically, no study has been conducted on the ovarian development of *F. taiwana* or its related species of the genus *Forcipomyia*. Current knowledge about ovarian development in dipterans is mostly based on studies on *Drosophila melanogaster* (fruit fly) and mosquitoes (reviewed in [11,12,13,14,15]). In general, the ovary of the female dipterans is composed of ovarioles of the polytrophic-meroistic type, in which the oocyte and nurse cells of an individual follicle are closely associated and enclosed by a single-layer of the follicular epithelium. Nurse cells and oocytes originate from oogonia, the female germline stem cells (GSCs), located in the germarium at the anterior-most region of the ovariole. In the germarium, oogonium divides to form the cystoblast, which undergoes several rounds of mitotic divisions with incomplete cytokinesis and produces interconnected cystocytes that subsequently differentiate into an oocyte–nurse cell complex. The channels between the oocyte and nurse cells, namely ring canals or intercellular bridges, are essential for the transportation of maternal materials, such as maternal mRNAs, ribosomes, and cytoplasmic organelles, from the nurse cells to the oocyte. In the posterior-most region of the germarium, the nascent oocyte–nurse cell complex is ensheathed by a monolayer of somatic follicle cells—the follicular epithelium—to form the primary follicle. When the primary follicle separates from the germarium, the oocytes begin to develop in the vitellarium region. According to the extent of the deposition of yolk in the oocytes, the oocyte developmental stages can be categorized in two sequential stages: previtellogenesis and vitellogenesis. During previtellogenesis, the chromosome of the oocytes condenses to form a karyosphere and that of the nurse cells becomes polyteny. Moreover, during this stage, the oocytes receive maternal materials from the nurse cells and obtain a limited amount of vitellogenin from the follicle cells. When the oogenesis transitions into vitellogenesis, the oocytes begin to absorb a substantial amount of vitellogenin synthesized by both the fat body and follicular epithelium. Therefore, the follicle during late vitellogenesis is densely filled with lipid droplets and yolk spheres.

Nuage is an amorphous, membrane-less, and electron-dense structure often observed either in proximity to nuclear pores or as large cytoplasmic accumulations in germ cells (reviewed in [16,17]). A series of ultrastructural studies on *Drosophila* have indicated the persistence of nuage in germ cells, although its expression in oocytes is gradually lost after oocyte specification [18,19,20]. Therefore, to identify germline cells in the germarium region or previtellogenic follicles, nuage could be a reliable marker. One of the major components of nuage is Vasa, which is a DEAD (Asp-Glu-Ala-Asp)-box RNA-helicase implicated in the translational control of maternal mRNAs [21,22,23]. The helicase core of Vasa exhibits RNA unwinding activity and is composed of an N-terminal DEAD-like helicases domain (DEXDc) and a C-terminal helicase superfamily domain (HELICc) [24,25]. Evidence reveals that the conformation of the helicase core, and not its RNA unwinding activity, is responsible for the localization of Vasa [22,26,27]. Functionally, Vasa is essential for the maintenance of nuage integrity and localization of nuage components, such as Aubergine, Krimper, and Maelstrom [28]. Recent studies have also suggested that Vasa, along with other nuage components (e.g., Argonaute-3, Aubergine, and Krimper), are required for piRNA biogenesis for repressing transposable elements in the female germline [28,29,30,31]. These findings suggest that Vasa is a critical nuage component and plays a central role in nuage-related biological functions.

Previous studies have not conducted a detailed examination of the ovarian development and oogenesis process for the genus *Forcipomyia*. Accordingly, in this study, we explored the ovarian structure and investigated early oogenesis in the hematophagous midge *F. taiwana*. During ovarian development, we observed a high degree of gonotrophic harmony—characterized by the synchronized development of oocytes—in *F. taiwana* following a blood meal. Additionally, the polytrophic follicle of *F. taiwana* was characterized as the single-nurse-cell type, which is the first to be reported in the family Ceratopogonidae. We also identified the perinuclear localization of the nuage in the germ line cells of *F. taiwana*. Moreover, we demonstrated that *F. taiwana* Vasa (FtVasa) could be localized to the nuage when expressed in the *Drosophila* germ line. This confirms that FtVasa is an authentic component of nuage. Therefore, our study provides a foundation for understanding the female reproductive physiology in the genus *Forcipomyia* and extends knowledge about oogenesis in hematophagous midges.

## 2. Materials and Methods

### 2.1. Female Midge Collection, Culture, and Ovary Dissection

The *Forcipomyia taiwana* females were collected by aspirator from the field in Hualien City, Taiwan. The collected female midges were identified according to the Catalogue and Keys of Chinese Ceratopogonidae (Insecta, Diptera) [32]. We dissected and examined more than 200 females right after the collection, and none of them had been observed with a developing ovary. The feeding of female midges was performed by using an artificial blood-feeding apparatus described previously [33]. The midges were cultured at 28 °C and relative humidity of 80%. At selected time points after blood-feeding, the ovaries were hand-dissected in phosphate-buffered saline (PBS) (130 mM NaCl, 7 mM Na_2_HPO_4_·2H_2_O, 3 mM NaH_2_PO_4_·2H_2_O, pH 7.4) for light microscopic examination, electron microscopic examination, or whole-mount immunostaining. For the analysis of ovarian development, one hundred of the females at selected time points after the blood meal were dissected for examining ovarian development.

### 2.2. Electron Microscopy

For scanning electron microscopy, female midges collected from the field were fixed with 2.5% (*w*/*v*) glutaraldehyde in 0.1 M cacodylate buffer and 1% tannic acid at 4 °C for 16 h. The fixed sample was extensively washed with phosphate buffer and dehydrated using a graded ethanol series. After critical-point drying and gold coating, the midges were examined using a scanning electron microscope (Hitachi S-4700, Hitachi, Tokyo, Japan).

For transmission electron microscopy (TEM), ovaries dissected from female midges were fixed with 2% paraformaldehyde and 2% glutaraldehyde in 0.1 M cacodylate buffer, pH 7.3, at 4 °C for 16 h. The post-fixation was performed with 1% OsO_4_ in 0.1 M cacodylate buffer at room temperature for 1 h. The ovaries were stained with 2% aqueous uranyl acetate for 1 h and subjected to series dehydration with alcohol and embedded in Spurr’s resin (Electron Microscopy Sciences, Hatfield, PA, USA). The ovaries were then sectioned using an ultramicrotome (Leica EM UC6, Leica, Wetzlar, Germany) and observed using a transmission electron microscope (Hitachi H-7500, Hitachi, Tokyo, Japan).

### 2.3. Whole-Mount Immunostaining

Hand-dissected ovaries of blood-fed midges were fixed in 4% formaldehyde for 20 min. After the removal of the fixative, fixed ovaries were washed three times in PBS containing 0.2% Tween 20 (PBST). Then, the ovaries were blocked with 2% bovine serum albumin in PBST for 1 h and incubated overnight with primary antibody diluted in PBS at 4 °C for 16 h. After the removal of the primary antibody solution, the ovaries were washed in PBST and incubated with a secondary antibody in PBST for 2 h at room temperature. Following three 30 min washes in PBST, the ovaries were mounted in anti-fade mounting solution. The following primary and secondary antibodies were used: rabbit anti-Vasa (1:100; a gift from Dr. Paul Lasko), rabbit anti-Krimper (1:500; a gift from Dr. Toshie Kai), goat-anti-rabbit Alexa Fluor 488 (1:200; Thermo Fisher Scientific, Waltham, MA, USA). F-actin was labeled by rhodamine-conjugated phalloidin (1:200; Sigma-Aldrich, St. Louis, MO, USA).

### 2.4. Cloning of FtVasa, Transgenes, and Drosophila Stocks

To clone the full-length coding sequence (CDS) of *F. taiwana vasa* (GenBank ID: MG662414.1), we performed a reverse transcription–polymerase chain reaction (RT–PCR). The total RNA from 30 pairs of ovaries of females at 40 h post-blood meal (PBM) was extracted by using the Quick-RNA Miniprep Kit (Zymo Research, Irvine, CA, USA). To perform the reverse transcription, 1 ug of the total RNA primed with oligo-(dT) primer was used for the 1st strand cDNA synthesis by using the SuperScript™ III First-Strand Synthesis SuperMix (Thermo Fisher Scientific, Waltham, MA, USA). The CDS of *F. taiwana vasa* was further PCR amplified by using the primer set (FtVasa-S: 5′ GGATCCATGGCAGATGAGTGGGATGACTTGG; and FtVasa-AS: 5′ ACTAGTGAATTCCTAGTACTCCCAGTCTTCTTCCTTCTCCA) and subcloned into the *pJET1.2/blunt* cloning vector (Thermo Fisher Scientific, Waltham, MA, USA). The CDS of *vasa* in *pJET1.2/blunt* was subsequently subcloned into *p{Pmat-tub67c:gfp}* vector, a *P*-element based vector containing maternal *tubulin 67c* promoter for the specific expression of GFP-FtVasa in female germline [34] by using restriction sites *Bam*HI and *Not*I. The final *p{Pmat-tub67c:gfp-Ftvasa}* plasmid and the helper plasmid encoding P-transposase were co-injected into the early embryos laid by *w^1118^*females (stock obtained from the Bloomington *Drosophila* Stock Center at Indiana University, USA). The detail procedures for generating transgenic flies were according to the standard protocol [35]. All the *Drosophila* stocks were raised at 25 °C on a standard cornmeal medium. Fly stocks used in Figure 8: P{*gfp-Dmvasa}* [27] and *P{gfp-Ftvasa}* (stock generated in this study).

## 3. Results

### 3.1. Synchronous Development of F. taiwana Follicles

**Figure 1:**
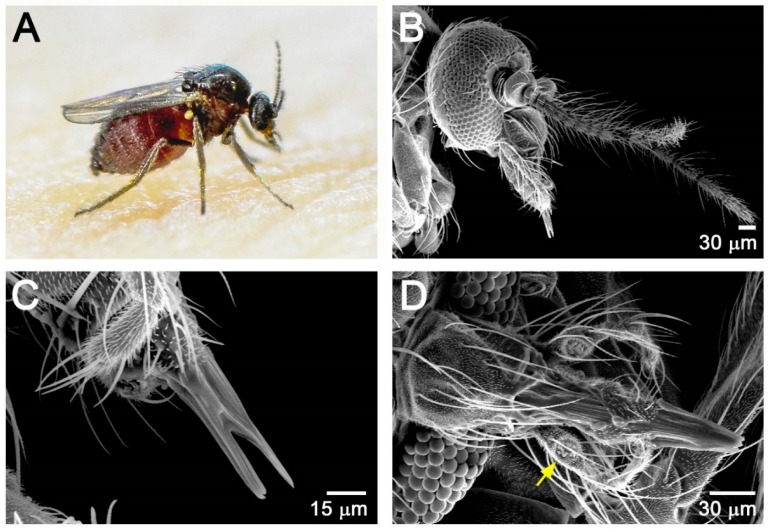
Morphology of female *F. taiwana.* (**A**) A blood-feeding female. (**B**,**C**) Scanning electron microscopy examination of head (**B**) and the short rasping-sucking mouthpart (**C**). (**D**) Dorsal view of the sucking-rasping mouthpart. Sensilla chaetica are located at a swollen pit (yellow arrow) of the third segment of maxillary palps.

The female *F. taiwana* has a black body with transparent and spotless wings measuring approximately 1.4 mm in length (Figure 1A). Its head is featured by contiguous compound eyes, a pair of long pilose antennae, a pair of maxillary palps that are swollen at the third segment containing sensilla chaetica, and a short rasping-sucking mouthpart (Figure 1B–D).

**Figure 2:**
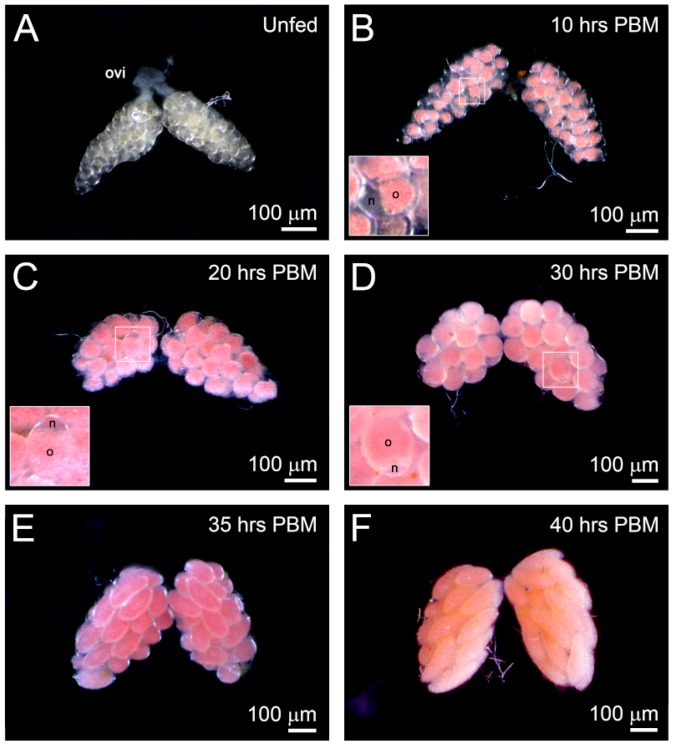
Morphological time course of the ovarian development of *F. taiwana* after a blood meal by light micrographs. (**A**) A pair of ovaries from an unfed female. The transparent ovaries consist of arresting primary follicles. (**B**–**F**) A pair of ovaries from females after a blood meal. (**B**) At 10 h post-blood meal (PBM), the insect shows the oocyte appear pink-colored with transparent nurse cells and follicular epithelium surrounding the oocyte. (**C**) At 20 h and (**D**) 30 h PBM, the follicular epithelium became thinner and invisible. (**E**) At 35 h PBM, the size of the ovary significantly increased and the nurse cell became barely visible. (**F**) At 40 h PBM, the follicles are approaching maturation. Insets: enlargement of developing follicles in the ovary. ovi: common oviduct; o: oocyte; n: nurse cell; PBM: post-blood meal. All females were maintained at 28 °C before dissection.

*F. taiwana* has an anautogenous reproductive strategy in which the female must have a blood meal to initiate ovarian development and egg maturation. To further analyze ovarian development pre- and post-blood meal (PBM) at 28 °C, ovaries of adult female midges were dissected at periodic intervals. We observed that the ovaries of unfed females were transparent and that the arresting primary follicles were barely observable through stereomicroscopy (Figure 2A). From 10 h PBM and onward, the ovary appeared pink, but the nurse cells and follicular epithelium remained transparent (Figure 2B, inset). From 20 to 30 h PBM, the follicular epithelium surrounding the oocyte became thinner and invisible. However, the crescent-shaped and transparent nurse cell remained identifiable posterior to the developing follicle (Figure 2C,D, insets). At 35 h PBM, the nurse cell was barely visible, and the size of the ovary was considerably increased (Figure 2E). At 40 h PBM, the follicles were about to mature (Figure 2F). The ovarian development process could be completed within 48 h PBM at 28°C. Our results reveal the synchronous development of *F. taiwana* follicles, which is a characteristic of gonotrophic harmony. Furthermore, the pink coloration of the developing oocyte is a prominent feature of ovarian development in *F. taiwana* (Figure 2B–F).

### 3.2. Ovarian Structure of F. taiwana

**Figure 3:**
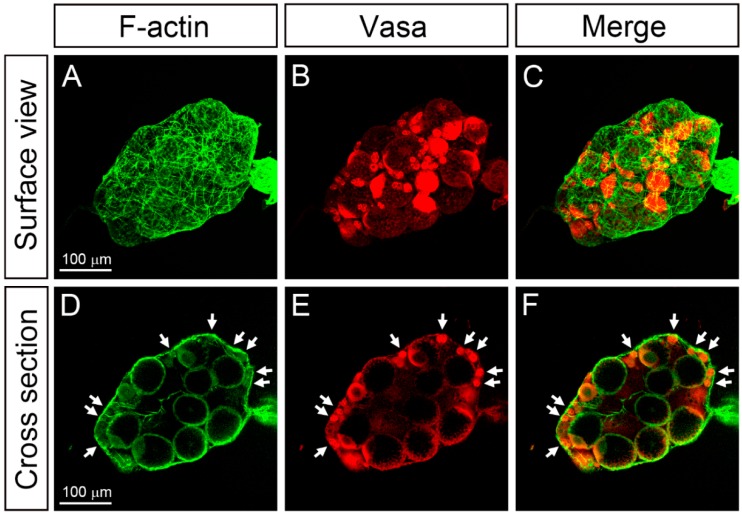
The ovarian structure and the alignment of ovarioles within the ovary of *F. taiwana.* (**A**–**F**) Confocal micrographs showing the ovarian structure of *F. taiwana* at 15 h post-blood meal. The F-actin was revealed by phalloidin staining (green), whereas the germline cells were marked by polyclonal rabbit anti-Vasa antibody (red). (**A**–**C**) The surface view revealed the F-actin enriched ovarian sheath (**A**) and germ line cells (**B**). (**C**) Merged image. (**D**–**F**) The optical cross-section showed that the germarium of each ovariole (arrows in E) was bound to the ovarian sheath (arrows in D). (**E**) Merged image.

To further analyze the ovariole organization inside the ovary, we used confocal microscopy to examine ovaries dissected from females at 15 h PBM. Fluorescein-5-isothiocyanate (FITC)-conjugated phalloidin that binds to the filamentous actin (F-actin) was used to reveal the cells within ovarioles and the actin filaments of muscle cells of the ovarian sheath. On the surface of the ovary, a layer of the ovarian sheath covering ovarioles was identifiable from the mesh-like network of the F-actin (Figure 3A,C). To reveal the germline cells within the ovarioles, we used the anti-Vasa antibody to mark the germline cells in the ovary (Figure 3B,E) because Vasa is highly conserved across the metazoans and robustly expressed in the germline cells. In the tangential optical section of the ovary, Vasa-positive germaria (Figure 3E, arrows) could be identified and were closely associated with the ovarian sheath that was intensely labeled with FITC-conjugated phalloidin (green color in Figure 3D,F).

**Figure 4:**
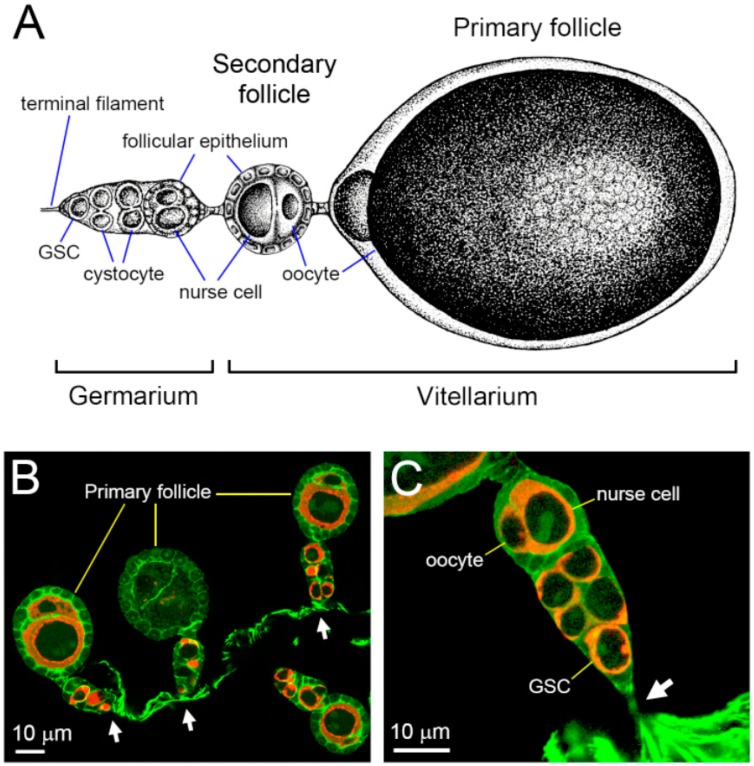
Schematic representation of the ovariole structure and its attachment to the ovarian sheath. (**A**) The ovariole of *F. taiwana* is composed of two regions: the germarium and vitellarium. In the germarium, it consists of a germline stem cell (GSC), germline cysts consisting two cystocytes, and a nascent follicle in which the oocyte and the nurse cell are enwrapped by a single-layer of follicular epithelium. The vitellarium contains at most two follicles: the primary follicle and secondary follicle. The secondary follicle emerges only when the primary follicle approaching its maturation. In the schematic representation, anterior is to the left. Terminal filament that anchors the ovariole to the ovarian sheath is indicated. (**B**) Ovarioles dissected from the ovary of an unfed female. Only primary follicles can be found. Germaria (arrows) from different ovarioles attached to the ovarian sheath separately. (**C**) Ovariole dissected from the ovary of a female at 15 h PBM. The ovariole is attached to the ovarian sheath via the terminal filament (arrow).

To further clarify the association of the germaria with the ovarian sheath, we separated the ovarian sheath and observed the connection of each germarium with the ovarian sheath at distinct positions of the terminal filament (Figure 4B,C, arrows).

### 3.3. Polytrophic-Meroistic Ovariole of F. taiwana

After the removal of the ovarian sheath, the polytrophic-meroistic ovariole of *F. taiwana* was revealed (Figure 4A–C). The ovariole could be divided into two developmental regions: the germarium and vitellarium (Figure 4A). The germarium was located at the anterior-most section of the ovarioles and contained female GSCs (Figure 4A,C). The vitellarium contained at most two follicles: a primary and a secondary follicle (Figure 4A). At the beginning of vitellogenesis, only one developing follicle, the primary follicle, appeared in the ovariole (Figure 4B). As the development of the primary follicle approached its maturation, the secondary follicle emerged from the germarium and was dormant in the previtellogenic phase (Figure 4A).

**Figure 5:**
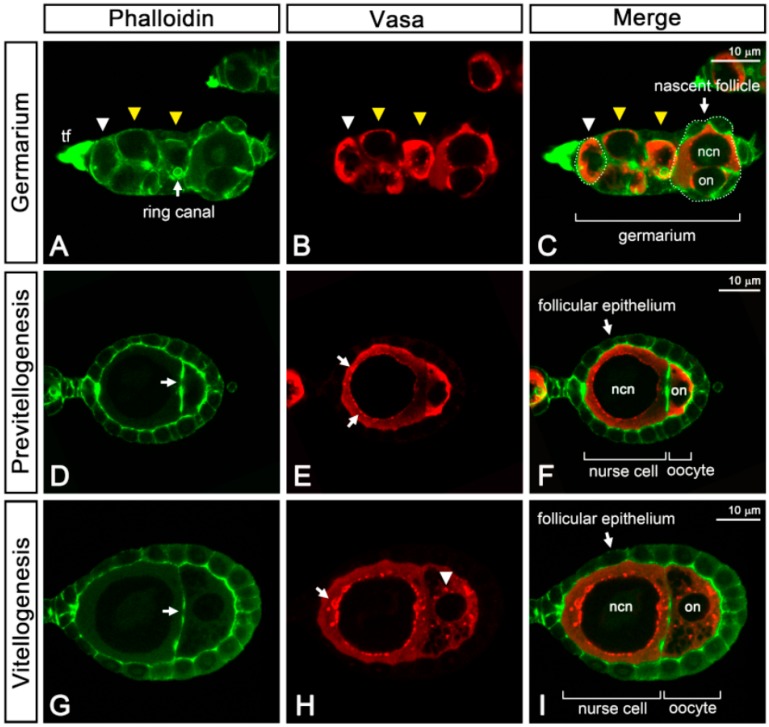
Ovariole structure and Vasa distribution during oogenesis. (**A**–**I**) Confocal micrographs showing the result of whole-mount immunostaining on ovarioles. The Phalloidin-conjugated FITC (green) and the anti-Vasa antibody (red) were used to visualize the cortical F-actin and germ line cells, respectively. Vasa specifically expressed in germline cells and enriched in the perinuclear regions of the nurse cells during vitellogenesis. (**A**–**C**) The germarium region. White arrowhead: germline stem cell, yellow arrowhead: cystocyte. The ring canal interconnecting cystocytes is indicated in (**A**). (**D**–**F**) The follicle at the previtellogenic phase from unfed females. Arrow in (**D**): ring canal; arrow in (**E**): perinuclear Vasa. (**G**–**I**) The follicle at the vitellogenic phase from females at six hours PBM. Arrow in (**G**): ring canal; arrow in (**H**): perinuclear Vasa; arrowhead in (**H**): Vasa aggregates in the ooplasm. ncn: nurse cell nucleus; on: oocyte nucleus; tf: terminal filament. For all the panels, anterior is to the left.

Using confocal microscopy, we observed GSC resides in the anterior-most region of the germarium (Figure 5A–C, white arrowheads). Posterior to the GSCs, multiple germline cysts containing two cystocytes were observed (Figure 5A–C, yellow arrowheads), which were interconnected through an actin-enriched cytoplasmic bridge, also known as the ring canal (Figure 5A, arrow). In the posterior-most region of the germarium, a nascent follicle (Figure 5C, white arrowhead) with two cystocytes, an oocyte, and a nurse cell could be observed. We further examined the nascent follicle using transmission electron microscopy.

**Figure 6:**
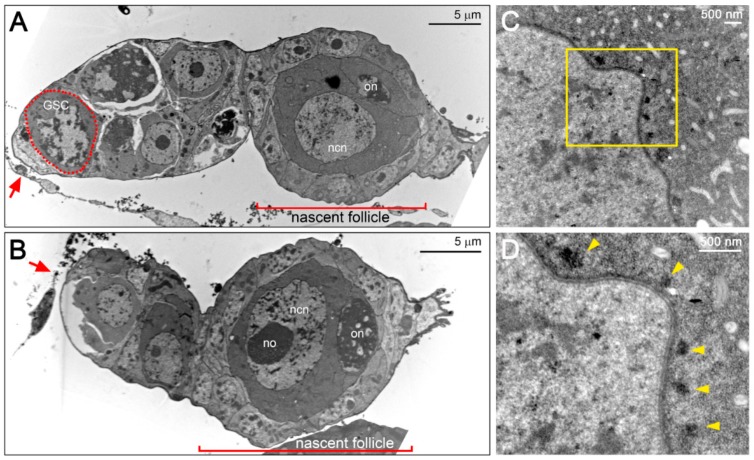
Transmission electron microscopy (TEM) examination of the germarium and the perinuclear nuage-like structure of the nurse cell. (**A**,**B**) TEM examination of germarium and nascent follicle. The germline stem cell (GSC) is marked by the dash-line. The oocyte is characterized by the condensed chromosomes at meiotic prophase. In nurse cell, the nucleus was featured by a large nucleolus and the patched chromatin. It is to be noted that the oocyte is moved from the lateral position (**A**) to the posterior end (**B**) of the nascent follicle. Red arrows indicate the ovarian sheath. (**C**,**D**) TEM examination of the perinuclear region in the nurse cell of a follicle at the previtellogenic stage. The electron-dense nuage-like structure is indicated by yellow arrowheads in (**D**). The region in the yellow rectangle of (**C**) is enlarged in (**D**). on: oocyte nucleus; ncn: nurse cell nucleus; no: nurse cell nucleolus. For all the panels, anterior is to the left.

The oocytes could be characterized by the condensed meiotic chromosomes, whereas the nurse cell contained patched chromatin and a distinct spherical nucleolus (Figure 6A,B). To form the nascent follicle, the oocyte–nurse-cell complex was enwrapped by a single-layer of follicular epithelium (Figure 5A–C and Figure 6A,B). In the nascent follicle, we observed a laterally positioned oocyte (Figure 5C and Figure 6A). Subsequently, the oocytes migrated to the posterior end of the follicle during maturation before leaving the germarium (Figure 6B).

In the previtellogenic phase, the oocyte diameter was no more than one-third of the nurse cell diameter (Figure 5D–F). The actin-rich ring canal connecting the only nurse cell and the oocytes was identified through phalloidin staining (Figure 5D, arrow). In the nurse cell, punctate Vasa signals began to emerge in the perinuclear cytoplasm (Figure 5E, arrows). After being subjected to the vitellogenic phase, the follicle was characterized by the enlargement of the oocyte and deposition of yolk granules in the ooplasm (Figure 5G–I). During this period, both the oocyte and the nurse cell increased in size, and the perinuclear aggregation of Vasa became increasingly apparent in the nurse cell (Figure 5H, arrow). In the ooplasm, Vasa protein aggregated and formed punctate structures (Figure 5H, arrowhead).

### 3.4. F. taiwana Vasa Could be Localized to the Perinuclear Nuage in Drosophila Nurse Cells

**Figure 7:**
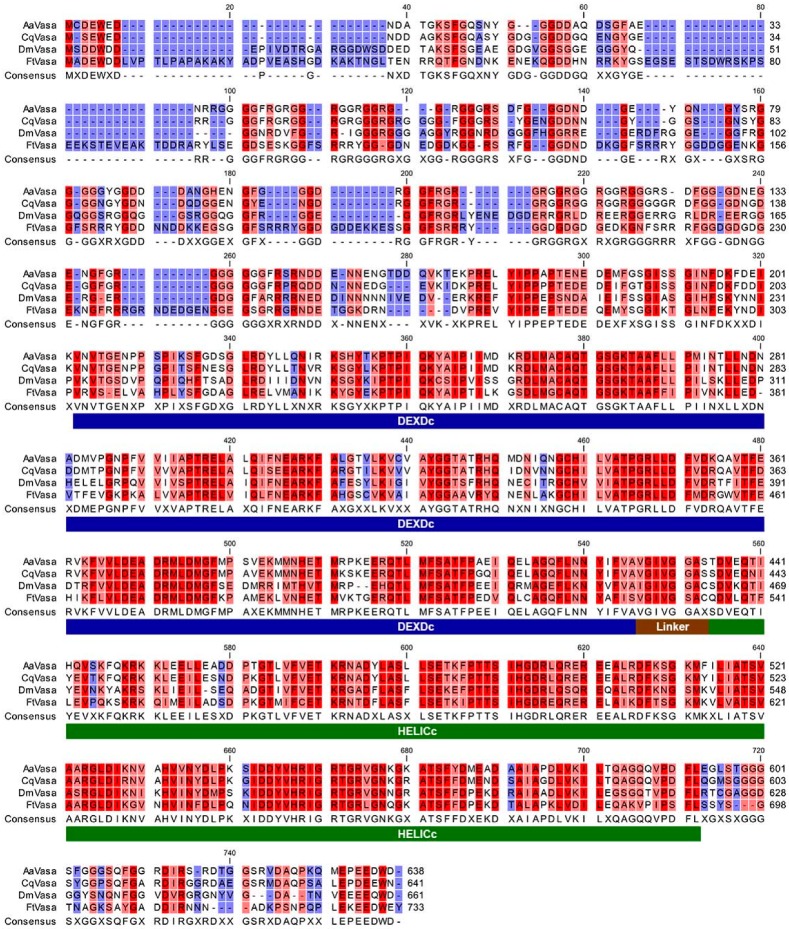
Sequence alignment of Vasa orthologs from *F. taiwana* and selected species. Multiple sequence alignment of Vasa orthologs from the biting midge *Forcipomyia taiwana* (FtVasa), fruit fly *Drosophila melanogaster* (DmVasa), southern house mosquito *Culex quinquefasciatus* (CqVasa), and yellow fever mosquito *Aedes aegypti* (AaVasa). The N-terminal region of FtVasa is less conserved. The C-terminal RNA helicase core domain, composed by DEXDc (blue bar) and HELICc (green bar), is highly conserved (see the main text for details). The gradient of the color reflects the level of sequence conservation in the alignment. Red: identical residues; pink: conserved residues; blue: residues with similar properties; white: not conserved residues.

Through confocal microscopy, we observed punctate Vasa staining around the nurse cell nucleus (Figure 5E,H, arrows). The perinuclear localization of Vasa was determined to be similar to that of the nuage identified in the *Drosophila* female germline. The nuage has been defined as amorphous and electron-dense structures located in the perinuclear region of nurse cells under electron microscopy [20,36]. To determine whether a nuage-like structure exists in the nurse cells of *F. taiwana*, we used transmission electron microscopy and observed electron-dense particles positioned adjacent to the nuclear membrane of the nurse cells (Figure 6C,D, arrowheads). Accordingly, we propose that the perinuclear electron-dense particles could be the counterpart of *Drosophila* nuage that carries germline determinants such as Vasa. To further investigate the molecular nature of FtVasa, we cloned its coding sequence through reverse transcription–polymerase chain reaction (RT–PCR) by using the total RNA extracted from the ovary (see Materials and Methods). We determined FtVasa to have 733 amino acids containing a divergent N-terminal extension and a C-terminal helicase core domain (Figure 7).

**Figure 8:**
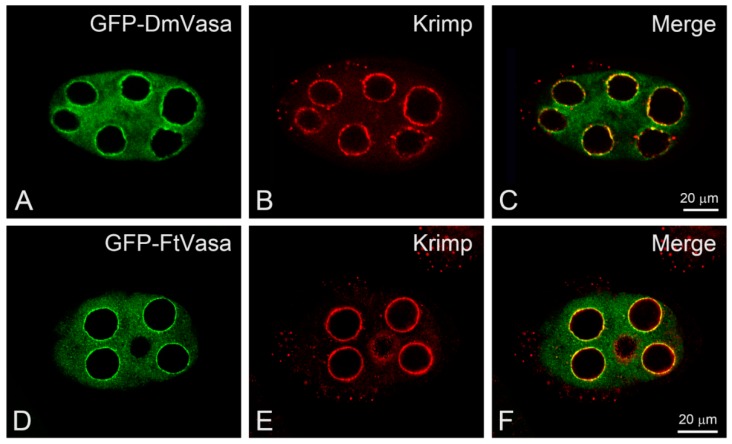
Localization of *F. taiwana* Vasa to the perinuclear nuage in *Drosophila* nurse cells. (**A**–**F**) Confocal micrographs showing the nurse cells of *Drosophila* egg chambers at stage 6. (**A**–**C**) (**A**) GFP-tagged *Drosophila* Vasa (GFP–DmVasa, green) is enriched in the nuclear membrane of nurse cells and colocalized with (**B**) nuage marker Krimper (Krimp, red). (**C**) Merged image. (**D**–**F**) (**D**) GFP-tagged *F. taiwana* Vasa (GFP–FtVasa, green) is colocalized with (**E**) Krimper (Krimp, red) in the nuage. (**F**) Merged image.

Although the N-terminal sequence of FtVasa is less conserved among Vasa proteins of dipteran species (Figure 7), the helicase core domain of FtVasa is highly conserved, sharing 76% similarity and 62% identity with that of the yellow fever mosquito *Aedes aegypti*, 74% similarity and 62% identity with that of the southern house mosquito *Culex quinquefasciatus*, and 74% similarity and 57% identity with that of the fruit fly *Drosophila melanogaster*. To further provide the evidence to show that FtVasa had the ability to localize to the nuage, we examined the localization of FtVasa in *Drosophila* nurse cells because the molecular marker of the nuage had not yet been identified in *F. taiwana*. Therefore, we generated transgenic flies expressing green fluorescent protein (GFP)-tagged FtVasa (GFP–FtVasa) in the female germline (see Materials and methods). By confocal microscopy, we can specifically observe the localization of GFP–FtVasa or the GFP-tagged *Drosophila* Vasa (GFP–DmVasa) [27], but not the endogenous *Drosophila* Vasa, by detecting the green-fluorescent emission out of the GFP tag. We, therefore, examined the colocalization between the nuage marker Krimp [28] and GFP–FtVasa to evaluate whether FtVasa could be localized to the nuage of *Drosophila* nurse cells. As a control, the GFP–DmVasa colocalized with Krimp (Figure 8A–C) at the perinuclear nuage in *Drosophila* nurse cells, as previously reported [27,28]. The colocalization of GFP–FtVasa with Krimp at the perinuclear region in the nurse cells was also observed in the *Drosophila* female germline expression GFP–FtVasa (Figure 8D–F). These results demonstrate that FtVasa could be targeted to *Drosophila* nuage and indicate that FtVasa is an authentic nuage component.

## 4. Discussion

In this study, we characterized the ovarian development, ovariole structure, and features of germline cells of the hematophagous midge *F. taiwana*. Knowledge about oogenesis in Ceratopogonidae is restricted to the genus *Culicoides* [9,10]; accordingly, this report extends current knowledge concerning ovarian development to the genus *Forcipomyia*. We observed that all follicles in the ovary of *F. taiwana* developed synchronously after a blood meal, signifying that *F. taiwana* exhibits a high degree of gonotrophic harmony, similar to most *Culicoides* midges [37]. However, in a few *Culicoides* species, the degree of gonotrophic harmony could be correlated with the completeness of the blood meal. For example, when *Culicoides barbosai* consumes a limited amount of blood, it shows a low degree of gonotrophic harmony [38]. Similar to *F. taiwana*, hematophagous mosquitoes belonging to the family *Culicidae* have a high degree of gonotrophic harmony after a complete blood meal [39,40]. By contrast, in the same *Culicidae* family, the nonhematophagous mosquito *Toxorhynchites rutilus* demonstrates no gonotrophic harmony [41]. Therefore, we hypothesized that in the suborder Nematocera, the size of blood meal could be correlated with the degree of gonotrophic harmony; moreover, hematophagous species would have a higher degree of gonotrophic harmony than that of nonhematophagous ones. Our observations in *F. taiwana* further support this hypothesis because *F. taiwana* consumes a complete blood meal if undisturbed and shows a high degree of gonotrophic harmony.

The same as other dipterans, the ovariole of *F. taiwana* is classified as a polytrophic-meroistic type. According to the position of germline cells revealed by Vasa staining, we observed that the germaria of individual ovarioles were separated from each other and dispersed all over the ovarian sheath. Such ovariole arrangement in the ovary of *F. taiwana* is similar to that of *Culicoides* midges and mosquitoes [10,42]. However, notably, the developing follicle of *F. taiwana* includes only one nurse cell, unlike *Culicoides* midges and mosquitoes, which have seven nurse cells instead of one. A single-nurse-cell follicle of a polytrophic ovariole has also been observed in the dark-winged fungus gnats (Sciaridae) and nonbiting midges (Chironomidae) [43,44]; therefore, it is not an exception in dipterans belonging to the suborder Nematocera. Apart from the dipterans, the earwig *Forficula auricularia* (Dermaptera: Forficulidae) also has a single-nurse-cell follicle originating from the two-cell cysts found in the germarium [43,45]. Considering the single-nurse-cell follicle has been identified only in the lower dipterans and dermapterans among a variety of polytrophic-meroistic insects of different orders, this raises an open question whether the single-nurse-cell follicle is an ancestral type of the polytrophic follicle. The number of nurse cells in the polytrophic follicle follows the 2^n^-1 rule, which is determined by the “n”th round of mitotic division of a cystoblast. In the two-cell cyst in the germarium of *F. taiwana*, we observed a ring canal connecting the oocytes and nurse cells. In other words, the cystoblast of *F. taiwana* divides only once and produces a two-cell cyst that subsequently differentiates into an oocyte–nurse cell complex in the germarium. Although the genetic control of cystoblast mitosis is unclear, developmental genetic studies on *Drosophila* have suggested that proteins required for the regulation of cyclin/cyclin-dependent kinase activity could play an essential role [46,47,48,49,50].

In germarium and previtellogenic follicles, we observed the accumulation of Vasa around the nurse cell nucleus as well as the existence of perinuclear nuage. Moreover, ectopic expression of FtVasa in the *Drosophila* ovary demonstrated that FtVasa could be localized to the nuage in the nurse cells. These results imply that FtVasa and *Drosophila* Vasa could share a similar mechanism in their perinuclear nuage localization. However, we did not observe a germ plasm localization of FtVasa in the *Drosophila* oocytes. In *Drosophila*, the localization of Vasa to the germ plasm requires the interaction between the HELICc domain of Vasa and the LOTUS domain of Oskar [27,51]. The HELICc domain is part of the conserved helicase core found in all Vasa orthologs, including FtVasa. Therefore, determining whether a LOTUS-domain-containing protein is expressed in the oocytes of *F. taiwana* for the recruitment of FtVasa to the germ plasm is appealing.

## 5. Conclusions

The hematophagous midge *F. taiwana* has a high degree of gonotrophic harmony after a blood meal. The oocyte shows a pink coloration during oogenesis and connects with a single-nurse cell. The follicle contains only one nurse cell, which is a distinct feature among hematophagous midges. Vasa, a germline marker, is localized in both the nurse cell and the oocyte. In nurse cells, the perinuclear localization of Vasa is reminiscent of the nuclear envelope-associated nuage observed by electron microscopy. By the ectopic expression of *F. taiwana* Vasa in the *Drosophila* germline, we showed that *F. taiwana* Vasa could be localized to the nuage in the nurse cells. This result suggests that *F. taiwana* Vasa is an authentic nuage component.
